# The effect of type 2 diabetes on electrocardiographic markers of significant cardiac events

**DOI:** 10.12669/pjms.343.14562

**Published:** 2018

**Authors:** Fatih Kuzu

**Affiliations:** 1 Fatih Kuzu, MD. Department of Endocrinology and Metabolism, Dumlupinar University, Evliya Celebi Training and Research Hospital, Kutahya, Turkey

**Keywords:** Arrhythmia, Electrocardiography, HbA1c, Type- 2 diabetes

## Abstract

**Background & Objectives:**

In diabetics, cardiac microvascular circulation disorder increases the risk of arrhythmia and sudden cardiac death. Tpeak-Tend (Tp-e) interval, Tp-e dispersion, and Tp-e/QT and Tp-e/QTc ratios measured by surface electrocardiogram (ECG) are new parameters used to evaluate ventricular arrhythmogenity. We aimed to compare QT dispersion (QTd), corrected QT dispersion (QTcd), P dispersion (Pd), Tp-e interval, Tp-e dispersion, and Tp-e/QT and Tp-e/QTc ratios in patients with type- 2 diabetes (T2D) with healthy population.

**Methods:**

Electrocardiographic parameters of a total of 100 patients diagnosed with T2D were retrospectively analyzed and compared with the ECG results of 100 healthy age-, sex-, and body weight and height-matched controls.

**Results:**

The Pd, QT interval, QTc interval, QTd, QTcd, Tp-e/QT, Tp-e/QT ratios were higher in the patients. A statistically significant correlation was found only between hemoglobin A1c and Tp-e dispersion, QTd, QTcd, Pd, Tp-e/QT and Tp-e/QTc parameters, in linear regression analysis. There was also a statistically significant positive correlation between *the values of low-density lipoprotein*, systolic blood pressure, and Tp-e dispersion.

**Conclusion:**

The risk of arrhythmia can be predicted by evaluating Tp-e interval, Tp-e dispersion, Tp-e/QT, and Tp-e/QTc ratio, suggesting heterogeneity of ventricular repolarization and P wave and Pd showing heterogeneity of atrial repolarization in diabetic patients.

## INTRODUCTION

Patients with diabetes mellitus have a high risk of cardiovascular disease and the cardiovascular complications are the leading cause of morbidity and mortality associated with diabetes.^1^ Type-2 diabetes (T2D) creates a prothrombotic state, leading to acute coronary syndromes by both endothelial damage and reducing antiaggregant factors, such as nitric oxide and prostacyclin and also increasing thrombotic substances such as fibrinogen, factor VII, and suppressing fibrinolysis by factors such as plasminogen activator inhibitors.^2^ Another important physiopathological process in diabetic patients is the glycosylation of *low-density lipoprotein* (LDL) particles. Along with glycosylation, LDL particles become more atherogenic. Dyslipidemia also contributes to the endothelial dysfunction. In addition, the expression of proinflammatory cytokines, such as serum interleukin-6, C-reactive protein and tumor necrosis factor-α is increased in T2D, leading to a proinflammatory condition. Coronary artery disease and arrhythmias caused by rapid developing atherosclerosis are major complications in this patients.^2^,^3^

In diabetic patients, the blood circulation in cardiac microvascular bed was disturbed by depending on autonomic neuropathy, prothrombotic, and proinflammatory state. This process increases the risk of arrhythmia, silent infarction and sudden cardiac death.^4^

The surface electrocardiogram (ECG) is often used in the evaluation of an increased arrhythmia risk. The measurements such as corrected QT interval (QTc), QTc dispersion (QTcd) and QT dispersion (QTd) in the surface ECG reveal the heterogeneity of cardiac repolarization. Also, these parameters are used to identify the risky patients which are associated with the increased risk of ventricular arrhythmia.^5^,^6^ Studies have also shown that the prolongation of QT interval and the increase in QTd are prognostic factors for the cardiac mortality in diabetic patients.^6^ The P dispersion (Pd) has been shown to be an independent risk factor for atrial fibrillation and atrial flutter.^7^,^8^ Tpeak-Tend (Tp-e) interval, Tp-e dispersion, Tp-e/QT and Tp-e/QTc measured in the surface ECG have recently played a part in the articles of the literature, and these are new parameters used to evaluate ventricular arrhythmogenity in many diseases. In studies in which the aforementioned parameters compared with those such as QTd, QTc, and QTcd, which were used for a relatively long time, they were shown to give as reliable results as at least these measurements for detecting impaired ventricular repolarization.^9^ The parameters considered to be markers for the risk of sudden cardiac death, such as QTd, QTc and QTcd are often affected by the heart rate.^6^ It has been shown that Tp-e interval, Tp-e/QT, and Tp-e/QTc rates can be used as a new marker in assessing ventricular repolarization and these parameters are even more reliable for evaluating the ventricular repolarization, as they are not affected by the changes of heart rate.^9^,^10^

In this study, we aimed to compare QTd, QTcd and Pd with Tp-e, Tp-e dispersion, Tp-e/QT and Tp-e/QTc as new markers in surface ECGs of T2D patients with those of healthy individuals.

## METHODS

Between June 2015 and June 2017, ECG results of 100 patients with T2D (60 females, 40 males, mean age 46.5±6.2 years; range: 29 to 58 years) who were followed in the endocrinology and metabolism diseases outpatient clinic of our hospital for T2D were retrospectively analyzed and compared with the ECG results of 100 healthy age-, sex-, and body weight and height-matched controls (66 females, 34 males, mean age 45.6±8.3 years; range: 19 to 68 years). Patients with the history of coronary artery disease, cerebrovascular disease and peripheral artery disease, valvular heart disease, atrial and ventricular arrhythmia, and those with cardiomyopathy, hypertension, chronic pulmonary disease, chronic liver disease, malignancies, acute or chronic renal insufficiency, thyroid dysfunction, electrolyte disturbance, anemia or other major disease were excluded from the study. Only the patients who underwent cardiac check-up during follow-up and those whose echocardiographic (ECHO) evaluations did not indicate valvular heart pathology and those with normal ejection fractions were included in the study. A written informed consent was obtained from each participant. The study was approved by the Ethics Committee for Clinical Researches of Dumlupinar University, Faculty of Medicine and conducted in accordance with the principles of the Declaration of Helsinki.

### Electrocardiography

All patients had sinus rhythm in ECG. The patients with findings suggesting ischemia on ECG, or branch block and atrioventricular conduction abnormalities, and those with the history of drug use which might affect ECG were excluded. In the study, 12-lead ECGs with the rate of 25 mm/s and voltage of 10 mm/mV were performed in the supine position of the cases. The P wave duration (PW) was determined as the intersection spot of the point where the P deflection separated from the isoelectric line and the isoelectric line of the last part of the P wave. Pd was calculated as the difference of the longest and shortest P-wave (Pd=Pmax-Pmin). The QT interval was determined the distance from the beginning of the QRS complex to the descending part of the T wave and the junction point of the isoelectric line. The QTc value was calculated according to the Bazett formula as QTc (milliseconds) = measured QT/√RR(seconds). QTd was calculated as the difference between the longest and shortest QT intervals (QTd=QTmax - QTmin), while QTcd was calculated as the difference between the longest QTc and the shortest QTc (QTcd=QTc max-QTc min). The Tp-e interval was determined as the distance from the peak of T wave to the descending part of the T wave and the junction of the isoelectric line. The Tp-e dispersion was calculated as the difference between the maximum and minimum Tp-e intervals. Tp-e/QT and Tp-e/QTc ratios were calculated by using these measurements.

### Laboratory analysis

The fasting blood sugar following the 10-12 hour night starvation, glycosylated hemoglobin (HbA1c), thyroid-stimulating hormones (TSH), free T4, total cholesterol, high-density lipoprotein (HDL) cholesterol, low-density lipoprotein (LDL) cholesterol, triglyceride, calcium, sodium, potassium and complete blood count were measured. Total cholesterol (TC), HDL cholesterol, LDL cholesterol and triglycerides (TG) were measured by an automatic analyzer (Beckman Coulter AU 2700). Fasting glucose level was determined by using the glucose hexokinase method (Beckman Coulter AU 2700). HbA1c values were determined by the method of high-performance liquid chromatography (TOSOH G7 device). TSH and free T4 were determined by immunoenzymatic method based on the principle of measuring chemiluminescence (Beckman Coulter DXI 600). Sodium, potassium and calcium were detected by ion selective electrode (Beckman Coulter AU 2700).

### Statistical analysis

Statistical analysis was performed using the SPSS version 18.0 program (SPSS Inc., Chicago, IL, USA). Descriptive data were expressed in mean ± standard deviation. The Kolmogorov-Smirnov test was used for normality analysis of the distribution of the numerical data. The Student’s t-test was used for normally distributed data, while the Mann-Whitney U test was used for the abnormally distributed data. In addition, the chi-square test was used to compare the categorical data. Stepwise multivariate linear regression model, including significant variables in univariate analyses was then used to determine which determinants independently explained a significant (p<0.05) fraction of the variance of the dependent variables. The data were analyzed at a 95% confidence level and considered significant at a *p*-value of less than 0.05.

## RESULTS

In our study, a total of 100 patients with T2D were compared with 100 healthy controls. The overall characteristics of the participants are summarized in Table-I. There was no significant difference between the two groups in terms of age, sex, height, weight, smoking, thyroid function tests, serum electrolytes, and blood count parameters (Table-I). As expected, HDL levels were significantly lower, while systolic blood pressure, diastolic blood pressure, LDL, total cholesterol, triglyceride, fasting glucose, and HbA1c values were significantly higher in the T2D patient group (Table-I).

**Table-I:** Demographic, clinical, and laboratory characteristics of patients and controls.

Variables	Patients (n=100)	Controls (n=100)	p
Age (years)^a^	46.5±6.2	45.6±8.3	0.37
Male/Female (n)	40/60	34/66	0.32
Height (cm)^a^	164.1±9.7	165.9±8.7	0.15
Weight (kg)^a^	82.5±15	80.8±17.5	0.47
Systolic blood pressure (mmHg)^a^	124±10.9	116.3±12	<0.001
Diastolic blood pressure (mmHg)^a^	73.7±9	69.7±8.7	0.002
Triglyceride (mg/dL)^b^	154(735-41)	122.5(398-36)	<0.001
Cholesterol (mg/dL)^a^	209.8±42.5	189.9±29.7	<0.001
LDL (mg/dL)^a^	128.7±34.5	116.4±25	0.01
HDL (mg/dL)^a^	43.7±8.1	47.1±10.1	0.01
Fasting glucose (mg/dL)^b^	164.5(103-456)	93.5(78-100)	<0.001
HbA1c (%)^b^	8.4(5.2-13.5)	5.35(4.5-6.1)	<0.001
TSH (uIU/mL)^a^	1.81±0.99	2.02±0.85	0.14
Free T4 (ng/dl)^a^	0.89±0.12	0.85±0.12	0.06
Hemoglobin (gr/dl)^a^	14.3±1.3	13.9±1.6	0.06
Hematocrit (%)^a^	43±3.1	42.1±3.4	0.08
Sodium (mmol/L)^a^	138.8±2.4	139.3±1.9	0.14
Potassium (mmol/L)^a^	4.46±0.33	4.44±0.30	0.53
Calcium (mg/dl)^a^	9.48±0.33	9.44±0.35	0.39
Smoker/non-smoker (n)	25/75	35/65	0.11

a Variables are expressed in mean ± standard deviation,

b Variables are expressed in median (min-max)

HDL - high-density lipoprotein, LDL - low-density lipoprotein,

TSH - thyroid stimulating hormone, HbA1c - Hemoglobin A1c.

The electrocardiographic parameters of both groups are shown in Table-II. The mean heart rate was significantly higher in the patient group (80.5±12.2 bpm) than in the healthy control group (74.6±10.1) (p<0.001). The P wave and P dispersion were significantly higher in the patient group than those in the control group (p<0.001 and p<0.001, respectively). QT interval, QTc interval, QTd and QTcd were significantly higher in the T2D group than those in the control group (p=0.01, p<0.001, p<0.001, p<0.001, respectively). Tp-e interval, Tp-e dispersion, Tp-e/QT and Tp-e/QTc values were significantly higher in diabetic patients than those in the healthy control group (p<0.001, p<0.001, p<0.001, p=0.01, respectively).

**Table-II:** Comparison of the electrocardiographic parameters of the groups.

Variables	Patients (n=100)	Controls (n=100)	P-value
Heart rate, (bpm)	80.5±12.2	74.6±10.1	<0.001
P wave (ms)	127.6±19.8	98.8±19.2	<0.001
QT interval (ms)	395.4±23.8	387.4±21.1	0.01
QTc interval (ms)	449.1±24.9	430.7±25.3	<0.001
Tp-e interval (ms)	101±17.5	87.4±18.7	<0.001
P dispersion (ms)	74.9±17.2	50.9±16.8	<0.001
QT dispersion (ms)	72.4±19.5	47.8±15.8	<0.001
QTc dispersion (ms)	89.4±29.8	53.2±19.3	<0.001
Tp-e dispersion (ms)	48.8±18.1	32.7±16.7	<0.001
Tp-e/QT	0.25±0.04	0.22±0.04	<0.001
Tp-e/QTc	0.22±0.04	0.20±0.04	0.01

ms: milliseconds, bpm: beat per minute, Parameters are expressed in mean ± standard deviation.

**Fig.1:**
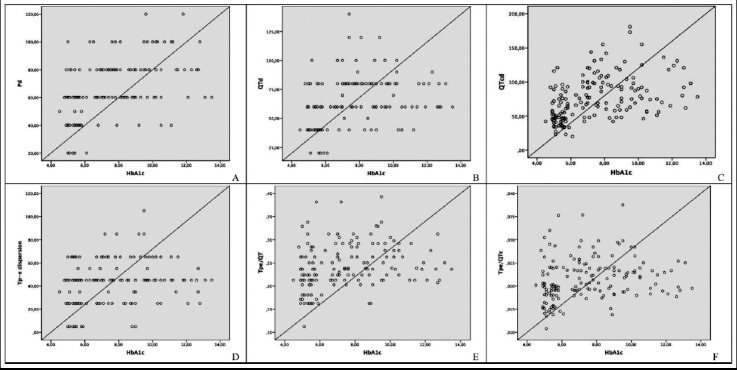
Correlation between hemoglobin A1c (HbA1c) and (A) P dispersion (B) QT dispersion (C) QTc dispersion (D) Tp-e dispersion (E) Tp-e /QT ratio (F) Tp-e/QTc ratio.

**Fig.2:**
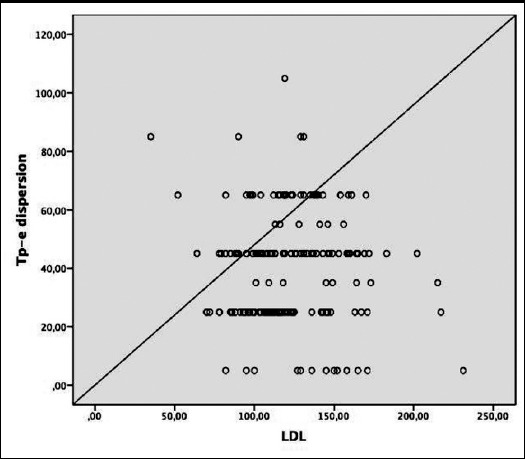
Correlation between low-density lipoprotein (LDL) and Tp-e dispersion.

**Fig.3:**
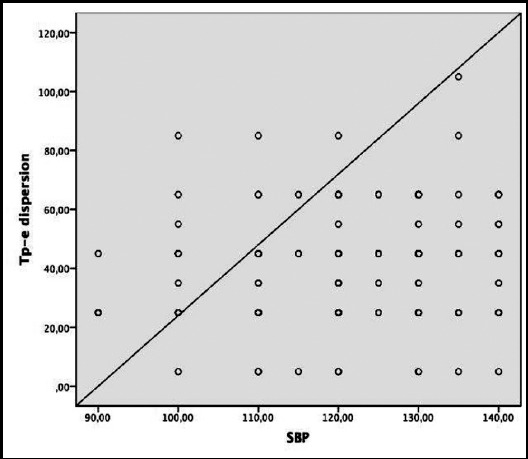
Correlation between systolic blood pressure (SBP) and Tp-e dispersion.

A statistically significant correlation was observed between HbA1c and Tp-e dispersion, QTd, QTcd, Pd, Tp-e/QT and Tp-e/QTc parameters in multivariate linear regression analysis (Table-III and Fig.1). There was also a statistically significant positive correlation between LDL and systolic blood pressure and Tp-e (Table-III, Fig.2, and Fig.3).

**Table-III:** Multivariate linear regression analysis of variable influencing electrocardiographic parameters in the patient and control groups.

	R^2^	B	95%	P-value

	Confidence Interval			
***Tp-e dispersion***				
HbA1c	0.09	0.33	0.15-0.42	<0.001
LDL	0.12	-0.21	-0.02 - 0.003	0.01
Systolic blood pressure	0.13	0.16	0.001-0.05	0.04
***QT dispersion***				
HbA1c	0.10	0.32	0.17-0.47	<0.001
***QTc dispersion***				
HbA1c	0.14	0.38	3.2-7.4	<0.001
***P dispersion***				
HbA1c	0.27	0.52	0.36-0.62	<0.001
***Tp-e/QT***				
HbA1c	0.06	0.25	0.002-0.009	0.001
***Tp-e/QTc***				
HbA1c	0.04	0.20	0.01-0.02	0.01

LDL: low-density lipoprotein, HbA1c: hemoglobin A1c.

## DISCUSSION

In many cardiovascular and non-cardiovascular diseases, QT interval, QTc, and QTd were shown to be increased than those in healthy subjects.^11^ It is well-known that diabetic autonomic dysfunction has important effects on the cardiovascular system and many studies have shown that QT interval, QTc and QTd were prolonged in these patients.^5^,^6^,^12^ Furthermore, it was suggested that there might be a relation between sudden cardiac deaths and QT prolongation in patients with diabetic autonomic neuropathy.^13^

In recent years, a number of studies have been conducted on reliability of aforementioned parameters in signaling ventricular arrhythmogenesis. Moreover, Tp-e interval, Tp-e dispersion, Tp-e/QT and Tp-e/QTc ratios as new parameters for transmyocardial repolarization have been shown to be superior to QT interval and QTd in predicting arrhythmia. However, they were also found to be more reliable in terms of being affected by heart rate and body weight.^9^,^14^ Among the events that cause the increase of atherosclerosis in diabetes mellitus are endothelial dysfunction, diabetic dyslipidemia, hypercoagulability, impaired fibrinolysis, increased platelet adhesion, oxidative stress, autonomic neuropathy and toxic effects of hyperglycemia.^15^ Although the pathophysiological mechanism is not fully established in diabetic patients, there is an increased risk of atrial fibrillation and ventricular arrhythmia, possibly due to structural abnormalities caused by prolonged hyperglycemia and increased fibrosis in atrial and ventricular myocardium.^16^,^17^

The formation of ventricular arrhythmias is explained by the presence of reentry circuits, triggered activity, and increased autonomy. Myocardial fibrosis, the cell loss in the living myocardial tissue and myocardial conduction pathways can create a favorable environment for the formation of micro-reentry circuits. Ventricular arrhythmias from simple ventricular extrasystole to fatal ventricular tachycardia may be triggered by the contribution of impaired electrical balance of the heart and the increased sympathetic system.^18^,^19^ In light of this information, it is important to evaluate the Tp-e interval, Tp-e dispersion, Tp-e/QT and Tp-e/QTc ratios, which are known to be associated with an increased risk of cardiac arrhythmia and which are relatively newly defined parameters of transmyocardial repolarization in diabetic patients.^17^,^18^ In this study, it was shown that Tp-e interval, Tp-e dispersion, Tp-e/QT and Tp-e/QTc ratios were longer in the T2D cases than those in the control group. In addition, in multivariate linear regression analysis there was a significant positive correlation between HbA1c and P dispersion, QTd, QTcd, Tp-e dispersion, Tp-e/QT and Tp-e/QTc ratios, while LDL level and systolic blood pressure were correlated with Tp-e dispersion.

In the study including fewer diabetic patients (n=43), Tokatli et al. showed that Tp-e interval, Tp-e/QT, Tp-e/QTc was higher in the patient group than those in the control group.^20^ In addition, there was a positive correlation between HbA1c, glucose and these parameters by Pearson correlation analysis. In our larger study in which we also added Tp-e dispersion, multivariate linear regression analysis showed that only HbA1c was a risk predictor of significant cardiac events. Besides, we investigated differently from this study, we also found a positive correlation between Tp-e dispersion, LDL and systolic blood pressure. In the study conducted by Clemente et al., only the Tp-e dispersion as new markers was investigated and it was found that this parameter was significantly higher in diabetic patients, consistent with our study.^21^

Diabetes mellitus has been shown to be an independent and potent risk factor for the development of atrial fibrillation.^22^ Pd is an important noninvasive indicator of intratrial conduction heterogeneity that forms a substrate for reentry, one of the pathophysiological mechanisms of atrial fibrillation.^23^,^24^ Both in T1D and T2D patients, it was detected that Pd was significantly higher in diabetic patients than those in the control group.^22^,^25^ In the study conducted by Dilaveris et al., the increase in Pmax and Pd was determined to be a highly specific and independent predictor of atrial fibrillation episodes.^23^,^24^ In our study, we found that the duration of PW and Pd was significantly increased in patients with T2D than those in the control group. In addition, the positive correlation between HbA1c and Pd was shown by multivariate linear regression analysis. In other words, as the HbA1c value increases, the Pd value increases proportionally and the risk of atrial fibrillation also increases.

### Limitations of the study

First, the parameters used in our study were measured manually. Although manual measurement has been accepted scientifically and many studies have been conducted by this method, it has been recognized that the measurement performed by a high resolution monitor with digital ECG recording gives much more accurate and standardized results in recent years. Another limitation is that normotensive patients with normal ECHO and ECG results were included in the study. As coronary angiography was not possible to perform for all patients in practice, subclinical ischemic heart disease might have been overlooked in the study group.

## CONCLUSION

Tp-e interval, Tp-e dispersion, Tp-e/QT and Tp-e/QTc ratio showing heterogeneity of ventricular repolarization with PW and Pd indicating heterogeneity of atrial repolarization are important electrocardiographic markers that should be evaluated in diabetic patients. These markers can be used to predict the risk of arrhythmia in diabetic patients. In addition, since these markers have significant correlation with HbA1c, the patients with high HbA1c values during the follow-up should be closely monitored in terms of important life-threatening cardiac events. Cardiologists should evaluate these patients in the early period and the detailed evaluation for antithrombotic, antihypertensive and antilipemic treatment should be started. In each control of outpatient clinic, ECG parameters showing the heterogeneity of cardiac repolarization should be evaluated until the HbA1c levels reach the target values.

## References

[1] Svensson AK, Svensson T, Kitlinski M, Almgren P, Engstrom G, Nilsson PM (2018). Incident diabetes mellitus may explain the association between sleep duration and incident coronary heart disease. Diabetologia.

[2] Kluppelholz B, Thorand B, Koenig W, Gala TH, Meisinger C, Huth C (2015). Association of subclinical inflammation with deterioration of glycaemia before the diagnosis of type 2 diabetes:the KORA S4/F4 study. Diabetologia.

[3] Beckman AJ, Creager AM, Libby P (2002). Diabetes and atherosclerosis:Epidemiology, pathophysiology and management. JAMA.

[4] Suys BE, Katier N, Rooman RP, Matthys D, De Beeck LO, Du Caju MV (2004). Female Children and Adolescents with Type 1 Diabetes Have More Pronounced Early Echocardiographic Signs of Diabetic Cardiomyopathy. Diabetes Care.

[5] Ninkovic VM, Ninkovic SM, Miloradovic V, Stanojevic D, Babic M, Giga V (2016). Prevalence and risk factors for prolonged QT interval and QT dispersion in patients with type 2 diabetes. Acta Diabetologica.

[6] Cox AJ, Azeem A, Yeboah J, Soliman EZ, Aggarwal SR, Bertoni AG (2014). Heart rate-corrected QT interval is an independent predictor of all-cause and cardiovascular mortality in individuals with type 2 diabetes:the Diabetes Heart Study. Diabetes Care.

[7] Li Z, Hertervig E, Carlson J, Johansson C, Olsson SB, Yuan S (2002). Dispersion of refractoriness in patients with paroxysmal atrial fibrillation:Evaluation with simultaneous endocardial recordings from both atria. J Electrocardiol.

[8] Ozdemir R, Guzel O, Kucuk M, Karadeniz C, Yılmaz U, Calik T (2016). The impact of 3:1 ketogenic diet on cardiac repolarization changes in children with refractory seizures:A prospective follow-up study. Neuropediatrics.

[9] Gupta P, Patel C, Patel H, Narayanaswamy S, Malhotra B, Green JT (2008). T(p-e)/ QT ratio as an index of arrhythmogenesis. J Electrocardiol.

[10] Ozdemir R, Isguder R, Kucuk M, Karadeniz C, Ceylan G, Katipoglu N (2016). A valuable tool in predicting poor outcome due to sepsis in pediatric intensive care unit:Tp-e/QT ratio. J Trop Pediatr.

[11] Okin PM, Devereux RB, Howard BV, Fabsitz RR, Lee ET, Welty TK (2000). Assessment of QT interval and QT dispersion for prediction of all-cause and cardiovascular mortality in American Indians. Circulation.

[12] Veglio M, Chinaglia A, Cavallo PP (2004). QT interval, cardiovascular risk factors and risk of death in diabetes. J Endocrinol Invest.

[13] Eranti A, Kerola T, Aro AL, Tikkanen JT, Rissanen HA, Anttonen O (2016). Diabetes, glucose tolerance, and the risk of sudden cardiac death. BMC Cardiovasc Disord.

[14] Shimizu M, Ino H, Okeie K, Yamaguchi M, Nagata M, Hayashi K (2002). T-peak to T-end interval may be a better predictor of high-risk patients with hypertrophic cardiomyopathy associated with a cardiac troponin I mutation than QT dispersion. Clin Cardiol.

[15] Selvin E, Marinopoulos S, Berkenblit G, Rami T, Brancati FL, Powe NR (2004). Meta-Analysis:Glycosylated Hemoglobin and Cardiovascular Disease in Diabetes Mellitus. Ann Intern Med.

[16] Mandala S, Di TC (2017). ECG Parameters for Malignant Ventricular Arrhythmias:A Comprehensive Review. J Med Biol Eng.

[17] Kato T, Yamashita T, Sekiguchi A, Sagara K, Takamura M, Takata S (2006). What are arrhythmogenic substrates in diabetic rat atria?. J Cardiovasc Electrophysiol.

[18] Piers SR, Everaerts K, Van der GRJ, Hazebroek MR, Siebelink HM, Pison LA (2015). Myocardial scar predicts monomorphic ventricular tachycardia but not polymorphic ventricular tachycardia or ventricular fibrillation in nonischemic dilated cardiomyopathy. Heart Rhythm.

[19] Qu Z, Weiss JN (2015). Mechanisms of ventricular arrhythmias:from molecular fluctuations to electrical turbulence. Annu Rev Physiol.

[20] Tokatli A, Kilicaslan F, Alis M, Yiginer O, Uzun M (2016). Prolonged Tp-e interval, Tp- e/QT ratio and Tp-e/QTc ratio in patients with type 2 diabetes mellitus. Endocrinol Metab.

[21] Clemente D, Pereira T, Ribeiro S (2012). Ventricular repolarization in diabetic patients:characterization and clinical implications. Arq Bras Cardiol.

[22] Bissinger A, Grycewicz T, Grabowicz W, Lubinski A (2011). The effect of diabetic autonomic neuropathy on P-wave duration, dispersion and atrial fibrillation. Arch Med Sci.

[23] Dilaveris PE, Gialafos EJ, Andrikopoulos GK, Richter DJ, Papanikolaou V, Poralis K (2000). Clinical and electrocardiographic predictors of recurrent atrial fibrillation. Pacing Clin Electrophysiol.

[24] Dilaveris PE, Gialafos EJ, Sideris SK, Theopistou AM, Andrikopoulos GK, Kyriakidis M (1998). Simple electrocardiographic markers for the prediction of paroxysmal idiopathic atrial fibrillation. Am Heart J.

[25] Yazici M, Ozdemir K, Altunkeser BB, Kayrak M, Duzenli MA, Vatankulu MA (2007). The effect of diabetes mellitus on the P-wave dispersion. Circ J.

